# Oxygen treatment reduces neurological deficits and demyelination in two animal models of multiple sclerosis

**DOI:** 10.1111/nan.12868

**Published:** 2023-01-10

**Authors:** Mario Amatruda, Kate Harris, Alina Matis, Andrew L. Davies, Daniel McElroy, Michael Clark, Christopher Linington, Roshni Desai, Kenneth J. Smith

**Affiliations:** ^1^ Department of Neuroinflammation UCL Queen Square Institute of Neurology London UK; ^2^ Department of Neurology Icahn School of Medicine at Mount Sinai New York New York USA; ^3^ Institute of Infection, Immunity, and Inflammation, College of Medical, Veterinary, and Life Sciences Glasgow Biomedical Research Centre Glasgow UK

**Keywords:** EAE, hypoxia, integrated stress response, neuroinflammation, oxidative damage, oxygen treatment

## Abstract

**Aims:**

The objective of the study is to explore the importance of tissue hypoxia in causing neurological deficits and demyelination in the inflamed CNS, and the value of inspiratory oxygen treatment, using both active and passive experimental autoimmune encephalomyelitis (EAE).

**Methods:**

Normobaric oxygen treatment was administered to Dark Agouti rats with either active or passive EAE, compared with room air‐treated, and naïve, controls.

**Results:**

Severe neurological deficits in active EAE were significantly improved after just 1 h of breathing approximately 95% oxygen. The improvement was greater and more persistent when oxygen was applied either prophylactically (from immunisation for 23 days), or therapeutically from the onset of neurological deficits for 24, 48, or 72 h. Therapeutic oxygen for 72 h significantly reduced demyelination and the integrated stress response in oligodendrocytes at the peak of disease, and protected from oligodendrocyte loss, without evidence of increased oxidative damage. T‐cell infiltration and cytokine expression in the spinal cord remained similar to that in untreated animals.

The severe neurological deficit of animals with passive EAE occurred in conjunction with spinal hypoxia and was significantly reduced by oxygen treatment initiated before their onset.

**Conclusions:**

Severe neurological deficits in both active and passive EAE can be caused by hypoxia and reduced by oxygen treatment. Oxygen treatment also reduces demyelination in active EAE, despite the autoimmune origin of the disease.

## Graphical abstract

Neuroinflammation can cause CNS hypoxia. The inflamed CNS of rats with experimental autoimmune encephalomyelitis (EAE, a model of multiple sclerosis) is hypoxic. Normobaric oxygen treatment of rats with EAE restores a normal CNS oxygenation and decreases the magnitude of both neurological deficit and histological signs of CNS damage. This finding demonstrates the role of tissue hypoxia to the pathobiology of neuroinflammatory diseases, and the potential value of oxygen as a therapy.

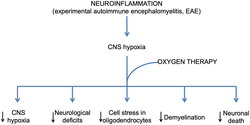

**Key Points**

- Severe, reversible neurological deficits can occur in neuroinflammatory diseases such as multiple sclerosis that cannot be explained by demyelination or neuronal damage; their cause is obscure.
- We explore the role of tissue hypoxia and treatment with oxygen in two animal models of MS: (a) *active* encephalomyelitis (EAE), which mimics many aspects of multiple sclerosis (MS) including severe neurological deficits, neuroinflammation, demyelination and neurodegeneration, and (b) *passive* EAE, where severe neurological deficits occur in the absence of demyelination and neurodegeneration.
- We report that in active EAE, oxygen treatment promptly reduces the neurological deficit and reduces demyelination, labelling for hypoxia, oligodendrocytes loss, and the integrated stress response in oligodendrocytes, without evidence of increased oxidative damage or changes in T‐cell infiltration and cytokine expression.
- In passive EAE, where the cause of the severe neurological deficits has hitherto remained uncertain because they occur in the absence of demyelination, we report that the spinal cord becomes hypoxic and that oxygen treatment significantly reduces the deficits if treatment is initiated before their onset.
- This study reveals the importance of tissue hypoxia in the pathobiology of both active and passive EAE, and the value of treatment by breathing raised oxygen.

## INTRODUCTION

Multiple sclerosis (MS) is an inflammatory demyelinating disease of the central nervous system (CNS), which causes debilitating neurological deficits in young adults [1]. The deficits can be caused by neuroinflammation in the absence of demyelination [2, 3], and increasing evidence from animal models suggests that in this case, the mechanism is a reduction in neuronal function due to tissue hypoxia arising from hypoperfusion [4, 5, 6, 7, 8, 9, 10, 11]. Certainly, hypoperfusion in MS has been recognised for over 70 years [12], and as methods to measure perfusion have become more sophisticated, the overwhelming finding is of very significant hypoperfusion in both relapsing–remitting (RRMS) and primary and secondary progressive MS (PPMS and SPMS) [7, 8, 9] that significantly correlates with disability [10, 11, 12, 13, 14, 15, 16, 17, 18]. Some studies have considered whether the reduction in cerebral blood flow is likely to be just a secondary consequence of reduced demand and cerebral atrophy, or whether it is a primary cause of damage, and the clear conclusion is that the reduction is a primary event probably causing pathology and thereby disability [7, 8, 14, 19, 20, 21, 22, 23, 24]. Hypoperfusion is likely to result in tissue hypoxia, and there is substantial evidence that the MS brain is actually hypoxic, including the expression of hypoxia‐related antigens [25, 26], the upregulation of genes induced by ischaemic preconditioning [25, 26, 27, 28] and the presence of lesions characterised by hypoxia‐like demyelination [27, 28, 29]. Most directly, using frequency domain near‐infrared spectroscopy to measure microvascular haemoglobin oxygen saturation in the brains of awake MS patients, Yang and Dunn have found a significant relationship between haemoglobin oxygen saturation and clinical disability, with 42% of saturation values more than twice the standard deviation lower than the control mean [23, 30].

The suspicion that hypoperfusion and hypoxia may be the link between inflammation and disability has been considerably strengthened by findings in a common animal model of MS, namely, experimental autoimmune encephalomyelitis (EAE), where hypoperfusion and tissue hypoxia correlate spatially, temporally and quantitatively with the expression of disability [4]. Mitochondrial function is also compromised [31, 32, 33, 34, 35, 36, 37, 38, 39, 40] apparently leading to an energy crisis that depolarises neurons/axons, preventing neurological signalling [31]. Convincingly, the hypoxia and resulting disability are promptly and significantly reduced by simple therapy to promote perfusion and oxygen delivery, using the vasodilating drug nimodipine, or promoting oxygenation by breathing raised oxygen [4, 5].

In this study, we investigate the effects of treatment with oxygen in two models of MS, namely both active and passive EAE. Active EAE (induced by immunising rats with recombinant myelin oligodendrocyte glycoprotein [rMOG]) mimics many aspects of MS including severe neurological deficits, neuroinflammation, demyelination, and neurodegeneration. In contrast, passive EAE (induced by administration of exogenous encephalitogenic T‐cells reactivated in vitro against MOG) does not involve the whole panoply of immune events involved in active autoimmunity, and although the neurological deficits can be severe, the mechanisms responsible have remained uncertain, in particular because they occur without signs of demyelination and neurodegeneration [41]. Furthermore, as the encephalitogenic response in passive EAE relies almost exclusively on the exogenous T‐cells, passive EAE represents an MS model in which the effects of oxygen therapy on neurological deficits can be investigated in a relatively simpler inflammatory environment.

Here, we explore the optimal timing and duration of oxygen treatment to reduce neurological deficits and demyelination in active EAE. We also explore the potential role of hypoxia in causing neurological deficits in passive EAE and find that the spinal cord is hypoxic (as in active EAE) and that breathing oxygen‐enriched ‘air’ reduces the magnitude of the neurological deficits. The absence of any significant effect of oxygen treatment on the magnitude of inflammation in active EAE, and the beneficial effect of oxygen in passive EAE in which the exogenous encephalitogenic T‐cells are unlikely to be modified once injected in the recipient animal, suggests that the oxygen treatment improved a metabolic defect rather than having an anti‐inflammatory effect. Notably, the beneficial effects of oxygen treatment occurred without exacerbating oxidative damage in the inflamed tissue.

## MATERIAL AND METHODS

### Animal models: Induction of active and passive EAE

Active or passive EAE was induced in female DA rats (8–9 weeks old, ~150 g, Harlan, UK) under general anaesthesia (2% isoflurane in air). Rats were kept in standard cages with food and water ad libitum on a 12‐h light–dark cycle.

*Active EAE* was induced by a subcutaneous immunisation at the base of the tail of 100 μg of rMOG (MOG_1–124_) in 1:1 (v/v) incomplete Freund's adjuvant (IFA). Control animals received an injection of IFA and saline emulsion alone.

*Passive EAE* was induced by the administration of encephalitogenic T‐cells reactive against MOG. Encephalitogenic T‐cells were isolated from donor DA rats sensitised to rMOG (active EAE) at Day 12 post‐immunisation. T‐cells were cultured for 1 day in T‐cell growth factor medium (complete DMEM + 15% horse serum + 15% MLA supernatant) then centrifuged, washed with fresh DMEM without serum, and co‐cultured with irradiated (30 Gy) thymocytes (10^8^ thymocytes with 2–4 × 10^6^ T‐cells) for 3 days in T‐cell re‐stimulation medium (complete DMEM + 1% rat serum + 10‐μg/ml rMOG). After 3 days the T‐cells were forming clusters, indicating proliferation and reactivation against the antigen (rMOG). Live T‐cells (irradiated thymocytes were dead by the third day in culture) were counted and suspended in Roswell Park Memorial Institute (RPMI) medium at 5 million T‐cells per millilitre. Five million reactive T‐cells (in 1 ml of RPMI medium) were injected intraperitoneally to induce passive EAE. Control rats received an injection of 1‐ml RPMI medium without cells.

### Behavioural tests

Animals were weighed daily, and the magnitude of neurological deficits was assessed using a 10‐point scale in which one point was given for each of the following neurological signs: tail tip weakness, tail weakness, tail paralysis, absence of toe spreading reflex, abnormal gait, left and right hindlimb paresis, left and right hindlimb paralysis, and moribund or dead. To assess the effects of treatment on function, a more detailed 30‐point scale was performed prior to, and following, treatment. A score of 0 to 2 points per hindlimb was given for each of the following functions: stretch withdrawal, toe spreading reflex, spasticity and plantar placement, and a score of 0 to 3 points was given for pinch withdrawal and nociception, together with tail paresis and paralysis (0–3 points), nociceptive response to tail pinch (0–3 points), flexion of the hip (0–2 points) and paralysis per forelimb (0–2 points). A score of zero indicated normal function. All behavioural assessments and analyses were performed blind.

### Oxygen treatment

Oxygen treatment was administered by placing the animals in a chamber (Biospherix, USA) with raised oxygen concentrations in air that was gently circulated. Control rats were kept in a similar chamber containing room air (21% oxygen). Carbon dioxide (≤0.04%) and humidity (40%–55%) were monitored.

#### Acute oxygen treatment in active EAE

Oxygen (95%, balance air) was administered for 1 h, and neurological function was assessed 1 h before raising the oxygen concentration, at the end of the oxygen treatment, and 1 h after returning the animals to room air.

#### Timing of the start of oxygen administration in active EAE

Oxygen (75%) was administered from either the first, second or third day of disease expression for 24 h. Animals were perfused after treatment and spinal cords were harvested for histopathological analyses.

#### Duration of oxygen treatment in active EAE

Oxygen (75%) was administered from the onset of the neurological deficit for either 24, 48 or 72 h. Animals were either perfused on the third day of disease (see Section 2.5) or returned to room air for the remaining duration of the experiment (up to 9 days from disease onset).

#### Prophylactic oxygen treatment in active EAE

Oxygen (75%) was administered continuously from the day of the immunisation up to 23 days post‐immunisation.

#### Therapeutic oxygen treatment in passive EAE

Oxygen (75%) was administered for either 24 or 48 h from the onset of the neurological deficit. Animals were then returned to room air and the progression of the disease was observed up to 4 days after the complete recovery of the neurological function.

#### Prophylactic oxygen treatment in passive EAE

Oxygen (75%) was administered continuously from the day of the injection of the encephalitogenic T‐cells and up to 4 days after the complete recovery of the neurological function.

### Analysis of tissue hypoxia: Pimonidazole and oxygen‐sensitive probe

Pimonidazole was injected intravenously via the saphenous vein under light general anaesthesia (1.5% isoflurane) 4 h prior to terminal fixation perfusion, as previously described [4, 5]. The magnitude of the tissue hypoxia was also assessed in vivo by the insertion of a physical oxygen‐recording probe into the dorsal horn of the spinal cord grey matter of rats under general anaesthesia (1.5% isoflurane) as previously described [4].

### Tissue processing and histology

Animals were intracardially perfused under general anaesthesia (2% isoflurane) with 0.1‐M phosphate‐buffered saline (PBS) containing heparin, followed by fixation with 4% paraformaldehyde in PBS. Spinal cord tissue was harvested and processed for cryosectioning. Immunohistochemistry was conducted on 12 μm‐thick sections using standard techniques, and a range of antibodies (Table S1). In all cases, analyses were performed on equally sized regions of interest from pictures taken under standardised conditions. All quantification was conducted using ImageJ software (NIH) and performed blinded to the treatment group. Quantification of HIF1α, MOG, ED1, LFB and pimonidazole was performed by calculating the percentage of the area labelled over the total white matter area, while E06 and 8OHdG were quantified by densitometric analysis, as previously described [42]. The neuronal count in the lumbar ventral horn was performed manually in spinal cord cross sections counting motor neurons stained with cresyl violet. Quantification of LFB, ED‐1, cresyl violet, and MOG was performed at five different levels of the spinal cord with a total of 20 sections per rat (sacral, lower lumbar, upper lumbar, lower thoracic and upper thoracic; four sections per level). In all other cases, quantification was performed in at least three sections of the lumbar spinal cord per animal. Cells positive for CC1, NeuN, p‐eIF2α, and CD3 were counted manually. The level of p‐eIF2α expression in neurons was calculated as pixel sum intensity (i.e., raw integrated density) normalised to the NeuN area. The number of neurons immunoreactive for p‐eIF2α was counted manually, normalised to the total number of neurons (labelled with NeuN) and expressed as a percentage. The area of CD3 positive clusters was calculated in ImageJ using the *freehand* selection tool. All immunofluorescent analyses were performed on DAPI‐positive cells.

### Quantitative real‐time polymerase chain reaction

Rats with active EAE were perfused on the third day from disease onset (peak of disease) in PBS‐heparin and the spinal cords were snap‐frozen on dry ice. RNA was extracted from 1‐cm frozen sections at the level of the lumbar spinal cord enlargement, using the TRIzol Plus purification kit (12183555). cDNA was synthesised using the Qiagen QuantiTect® Reverse Transcription Kit following the manufacturer's instructions. Primers (Table S2) were designed using the NCBI Primer Designing Tool, tested with BLAST (http://blast.ncbi.nlm.nih.gov/Blast.cgi), and purchased from Integrated DNA Technologies (UK). Real‐time polymerase chain reaction (PCR) was performed using 1X SYBR Green master mix (Applied Biosystems, USA) using standard techniques.

### Statistical analysis

GraphPad Prism v9.0 (USA) was used for statistical analyses and graph generation. All data were tested for Gaussian distribution using the Shapiro–Wilk test of normality. Parametric or non‐parametric statistical tests were used respectively for comparison of datasets that were normally or not normally distributed. All statistical analyses were two‐tailed and considered significant when *p* < 0.05. For normally distributed datasets, parametric tests were used as follows: independent Student's *t* test was used when two groups were compared; one‐way ANOVA test was used to compare multiple groups, with Tukey's post hoc test to compare all groups with each other. For datasets that were not normally distributed, the non‐parametric Mann–Whitney *U* test was used to compare two groups of unmatched samples, while the Kruskal–Wallis one‐way ANOVA followed by Dunn's multiple comparison test was used when comparing more than two groups. Differences in EAE disease severity over time were assessed using non‐parametric tests: the Friedman's repeated measures test with Dunn's multiple comparison post‐test was used to compare more than two groups, while the Wilcoxon matched‐pairs signed rank test was applied when comparing two groups. All data are shown as mean ± standard error of the mean (SEM).

## RESULTS

### Treatment with oxygen for 1 h transiently improves neurological deficits in rats with EAE

**FIGURE 1:**
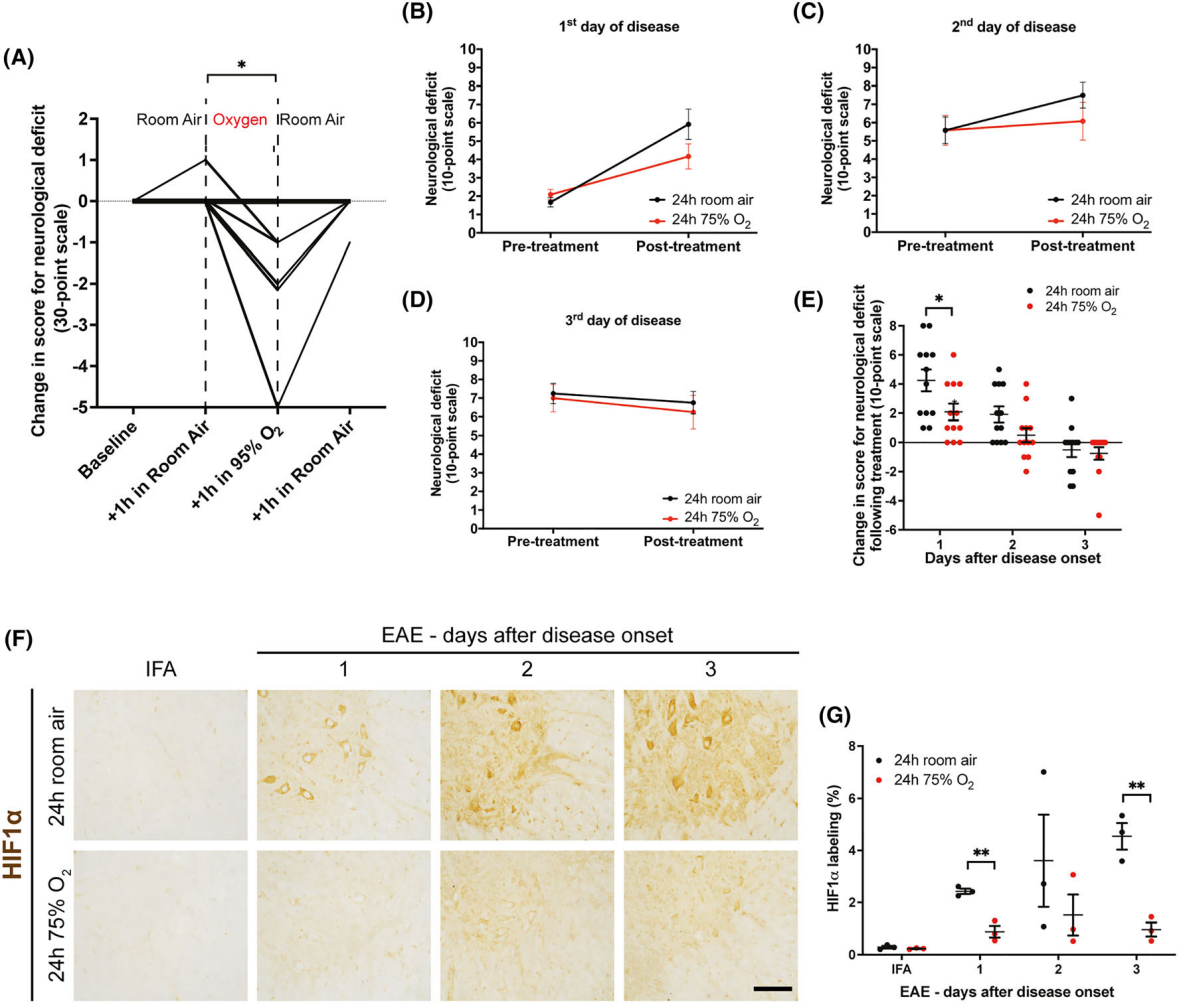
Effects of treatment with oxygen for 1 or for 24 h, in rats with active experimental autoimmune encephalomyelitis (EAE). (A) Exposure to 95% oxygen for 1 h promptly improved neurological function (assessed using a 30‐point behavioural test) in 50% of treated rats with active EAE at different levels of disease severity (*n* = 3 with impairment of tail motility, *n* = 3 with tail paralysis and hind limb paresis, and *n* = 4 with tail and hind limb paralysis). Lines represent *changes* in neurological score for single animals. Positive and negative scores represent an exacerbation or an improvement of neurological deficits respectively. (B–D) Disease progression in rats with active EAE treated with 75% oxygen (or room air) for 24 h on either the first, second, or third day of disease. (E) Graph showing the change in score for neurological deficit after 24 h of 75% oxygen, calculated as the difference between the absolute score before and after the treatment. Rats with EAE treated with oxygen on the first day of disease showed a significantly reduced exacerbation of the neurological deficits compared with time‐matched controls. Negative values represent improvements of neurological function. (F) Images showing a portion of the ventral horn of spinal cords labelled for HIF1α. Increased HIF1α labelling was observed in rats with active EAE compared with incomplete Freund's adjuvant (IFA) controls, and the labelling was stronger on the second and third day of disease: Labelling was particularly prominent in motor neurons. Rats with active EAE treated with oxygen for 24 h on either the first, second or third day of disease showed reduced levels of HIF1α compared with animals maintained in room air. Scale bar = 200 μm. (G) Quantification of HIF1α immunolabelling shows that HIF1α expression was significantly decreased in rats with active EAE exposed to oxygen for 24 h on the first and third day of deficits, compared with time‐matched room air controls. Statistical differences for Parts (A) and (E) were determined using the two‐tailed Mann–Whitney *U* test, statistical differences for G was determined using the two‐tailed Student's *t* test. (A) *N* = 10. (B–E) *N* = 12 per group. (G) *N* = 3 per group.

Ten animals with different levels of neurological deficit during the first peak of the disease (*n* = 3 with impairment of tail motility, *n* = 3 with tail paralysis and hindlimb paresis, and *n* = 4 with tail and hindlimb paralysis) were treated with oxygen‐enriched (95%) air for 1 h. During the first hour in room air, one animal with EAE exhibited an exacerbation of its neurological deficit by one point (Figure 1A), but, following treatment with oxygen during the subsequent hour, 50% of the animals (1 mild, 1 moderate and 3 severe) exhibited improvements in tail and hindlimb motility, and/or nociceptive perception assessed as vocalisation response to pinches at different levels of the tail (tip, middle and base of the tail) (Figure 1A). Interestingly, the neurological deficits returned when animals were placed back into room air for the next hour (Figure 1A).

### The timing of the start of oxygen administration affects the magnitude of the therapeutic benefit

As oxygen administration for 1 h resulted in a prompt, but transient improvement of the neurological deficits, we investigated whether longer exposure to oxygen could have a greater or more prolonged, beneficial effect on the disease course. To diminish any possible side effects caused by prolonged exposure to oxygen at high concentrations (e.g., 95%), we lowered the level of oxygen in the inspired air to 75%, a concentration that we have previously shown to be sufficient in restoring a normal oxygen partial pressure in the spinal cord of animals with active EAE [4]. To explore the optimal time for treatment to commence, animals with active EAE were randomly assigned to receive 75% oxygen, or room air, for 24 h, starting on the first, second or third day of disease expression (*n* = 72; 12 per group). Interestingly, oxygen treatment appeared to attenuate the neurological deficit irrespective of the day on which the treatment started (Figure 1B–E); however, the beneficial effects were statistically significant in animals treated on the first day of deficit (*p* = 0.03) compared with time‐matched controls treated with room air (Figure 1B,E).

### Oxygen treatment for 24 h decreases spinal cord hypoxia independently of the time at which treatment is started

Spinal cord sections from animals with active EAE, treated with 75% oxygen on the first, second or third day of deficit, were examined immunohistochemically for HIF1α to determine whether 24 h of oxygen treatment at different stages of active EAE could reverse tissue hypoxia. Spinal cords from animals injected with adjuvant alone (IFA controls), and animals with EAE treated with room air, served as controls. Significant HIF1α labelling was evident in the spinal cord of animals with active EAE. In particular, HIF1α immunoreactivity increased with the progression of the disease and was the most intense 3 days post‐onset of neurological deficit (Figure 1F). No such labelling was evident in IFA controls (Figure 1F). Notably, labelling for HIF1α was decreased following 24 h of oxygen, irrespective of day on which the treatment started (Figure 1F,G), but in particular on the first and third day of disease (both *p* = 0.003) (Figure 1G).

### Duration of oxygen treatment: 24, 48 and 72 h of oxygen exposure from the onset of deficits

**FIGURE 2:**
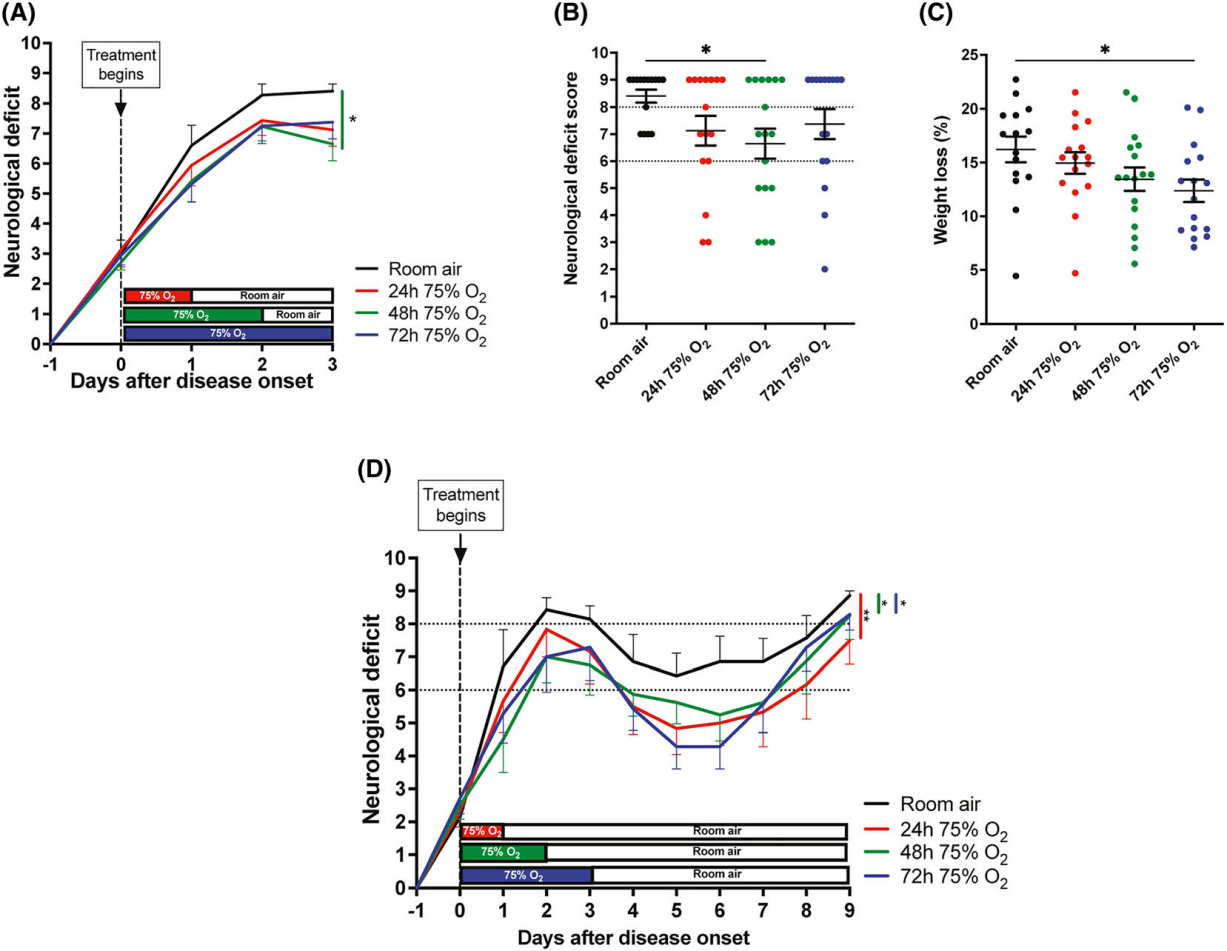
Effect of treatment with oxygen for 24, 48 or 72 h from the onset of neurological deficits. (A) Rats with active experimental autoimmune encephalomyelitis (EAE) were exposed to oxygen (75%) from the onset of disease (Day 0; vertical dashed line) for either 24, 48 or 72 h. Rats treated with oxygen for 24 and 48 h were returned to room air up to the third day of disease. Rats treated with oxygen showed a less severe progression of neurological deficits which was significant in rats treated for 48 h compared with control animals breathing room air. Data were compared using the repeated measurements Friedman's one‐way ANOVA with Dunn's multiple comparisons test. (B) Score for neurological deficit at the peak of disease (3 days after onset) shows that 50% of rats treated with oxygen did not develop hindlimb paralysis (score <8), with some maintaining the ability to walk using the hindlimbs (score <6). In contrast, all rats in the room air control group lost the ability to walk using the hindlimbs (score >6), and the majority of them (75%) developed hindlimb paralysis (score ≥8). Rats treated with oxygen for 48 h showed significantly less neurological deficit at the peak of disease compared with controls. Kruskal–Wallis test with Dunn's multiple comparisons. (C) Graph showing the percentage of body weight lost by rats with active EAE at the third day of disease expression relatively to the body weight at the onset of deficits. Rats treated with oxygen lost less weight than room air controls, and significantly less when treated for 72 h. One‐way ANOVA with Dunnet's multiple comparisons test. (A–C) Data show results plotted from two independent EAE experiments with at least six animals per group. Total *n* numbers: room air, *n* = 15; 24 h 75% O2, *n* = 16; 48 h 75% O2, *n* = 17; 72 h 75% O2, *n* = 16. (D) Graph showing the progression of disease in rats with active EAE exposed to oxygen (75%) for 24, 48 or 72 h from the onset of neurological deficits. After treatment, rats were returned to room air, and the neurological deficits were monitored for up to 9 days after the onset of deficits. All durations of oxygen exposure ameliorated disease progression compared with room air controls, and the beneficial effect persisted for several days after the cessation of treatment. Rats treated with oxygen experienced a more pronounced remission of the neurological deficits after the first peak of disease, recovering the ability to walk using the hindlimbs (score <6). Statistical differences in the overall disease progression over time were determined using the repeated measurements Friedman's one‐way ANOVA with Dunn's multiple comparisons test. Room air, *n* = 7; 24 h 75% O2, *n* = 6; 48 h 75% O2, *n* = 8; 72 h 75% O2, *n* = 7.

To explore the effect of duration of oxygen treatment, animals with active EAE were exposed to oxygen (75%) for either 24, 48 or 72 h from the onset of neurological deficits (*n* = 49, minimum 16 animals per group). Oxygen treatment ameliorated the severity of disease progression in all groups compared with control rats kept in room air (*n* = 15), achieving significant protection in animals treated for 48 h (*p* = 0.04) (Figure 2A). At the first peak of disease (three days after disease onset), animals treated with oxygen for 24, 48 or 72 h exhibited a milder neurological deficit compared with room air‐treated controls (Figure 2B), but particularly in the group treated with oxygen for 48 h (*p* = 0.02). Further, approximately 50% of animals in all oxygen‐treated groups did not develop hindlimb paralysis (score <8), and some rats maintained the ability to walk (score <6) throughout (Figure 2B). In comparison, all animals in the control group exposed to room air lost the ability to walk (score >6), with 75% showing hindlimb paralysis (score ≥8) (Figure 2B).

Body weight progressively decreases from the day of onset of neurological deficit in animals with EAE and it is a marker of disease activity [43]. Animals with active EAE treated with oxygen exhibited a milder loss of body weight compared with control rats breathing room air (Figure 2C), particularly in the group treated with oxygen for 72 h (*p* = 0.04).

To assess the progression of the disease following treatment with oxygen, animals were kept in standard cages breathing room air up to the second peak of neurological deficits (*n* = 28). In animals that were returned to air, the beneficial effect of oxygen was found to persist for several days after the termination of treatment, even when oxygen was applied for only the first 24 h of disease expression (Figure 2D). Indeed, in all the groups treated with oxygen the average score for neurological deficit was always lower than the room air control (room air vs. 24 h 75% O_2_, *p* = 0.009; room air vs. 48 h 75% O_2_, *p* = 0.0499; room air vs. 72 h 75% O_2_, *p* = 0.0314), with the rats treated for 72 h showing the most pronounced recovery of neurological function following the first attack of disease (Figure 2D). However, although oxygen administration at the onset of disease attenuated the overall severity of deficits, providing a better and longer recovery during the remitting phase, it did not prevent the development of the second peak of neurological deficits (Figure 2D).

### Neuropathological characterisation of rats with active EAE treated with oxygen

We performed histological examinations for signs of EAE pathology (oligodendrocyte loss, demyelination, cell stress, oxidative damage and inflammation) in spinal cord sections taken on the third day of disease (peak of deficits) from rats exposed to oxygen from disease onset for either 24, 48 or 72 h. Rats with active EAE treated with room air and healthy (IFA) controls were used for comparison.

#### Oxygen treatment protects the spinal cord of rats with active EAE from oligodendrocyte loss and demyelination

**FIGURE 3:**
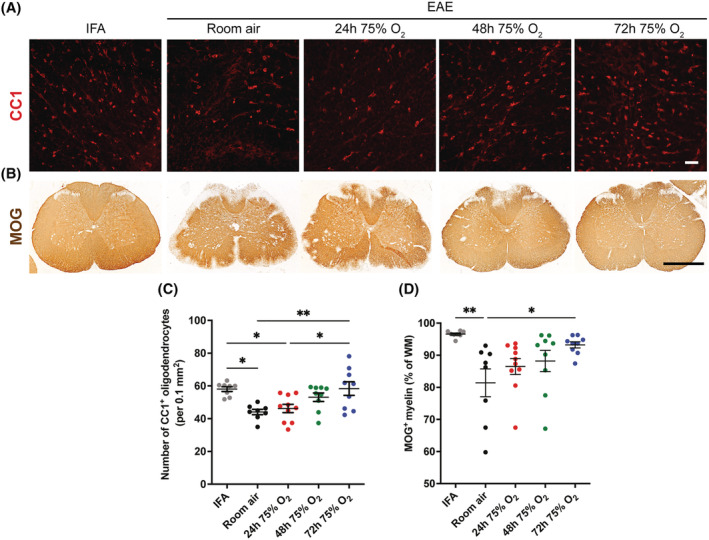
Histological examination of the number of oligodendrocytes and myelination of the white matter in the spinal cord of rats with active experimental autoimmune encephalomyelitis (EAE) treated with oxygen. (A–B) Images of the lumbar spinal cord showing immunofluorescence for CC1 (red, oligodendrocytes) in the dorsal column (A) or immunolabelling for MOG (brown, myelin) (B) of incomplete Freund's adjuvant (IFA) controls and EAE rats at the peak of disease. Rats with active EAE were either exposed to room air or treated with oxygen from the onset of disease, for 24, 48 or 72 h. Absence of MOG labelling in Part (B) reveals regions of demyelination. Scale bar is 20 μm in Part (A) and 1 mm in Part (B). (C) Counts of cells positive for CC1 at the peak of disease reveal that the number of oligodendrocytes is significantly decreased in rats with active EAE breathing room air, compared with IFA controls. Treatment with oxygen protected oligodendrocyte numbers in proportion to the duration of the treatment, and significantly in rats exposed to oxygen for 72 h compared with room air controls. (D) Quantification of immunohistochemistry for MOG in animals with active EAE confirmed that myelin labelling was reduced in the white matter (WM) of rats with EAE compared with healthy (IFA) controls. Reduced MOG immunoreactivity was less prominent in rats treated with oxygen compared with room air controls in proportion to the duration of the treatment, and significantly in rats treated with oxygen for 72 h. Statistical differences for Parts (C) and (D) were determined using the one‐way ANOVA with post‐hoc Tukey's multiple comparisons test. IFA, *n* = 8; room air, *n* = 8; 24 h 75% O2, *n* = 10; 48 h 75% O2, *n* = 9; 72 h 75% O2, *n* = 9.

The number of CC1‐positive mature oligodendrocytes in the spinal cord (Figure 3A) of rats with active EAE breathing room air was decreased by 24% compared with IFA controls (Figure 3A,C; IFA vs. room air, *p* = 0.011), but treatment with oxygen resulted in an increased survival of oligodendrocytes, correlating with the duration of oxygen exposure (Figure 3A,C). Treatment for 72 h resulted in a significantly greater number of CC1‐positive oligodendrocytes compared with room air controls (room air vs. 72 h 75% O_2_, *p* = 0.007) and with rats treated with oxygen for 24 h (24 h 75% O_2_ vs. 72 h 75% O_2_, *p* = 0.019). In fact, animals treated with oxygen for 72 h showed a similar number of CC1‐positive oligodendrocytes to the number in IFA controls (Figure 3C). The protection of oligodendrocytes was accompanied by a protection from myelin loss, as assessed by immunolabelling for MOG (Figure 3B). Indeed, myelination of the white matter was significantly reduced in rats with EAE exposed to room air compared with IFA controls (*p* = 0.0042) but not in animals exposed to oxygen, which showed a decreased loss of MOG‐positive myelin in proportion with the duration of the treatment (Figure 3B,D), achieving statistical significance with 72 h of treatment (Figure 3D; room air vs. 72 h 75% O_2_, *p* = 0.031).

#### Oxygen treatment decreases the integrated stress response in oligodendrocytes in rats with active EAE

**FIGURE 4:**
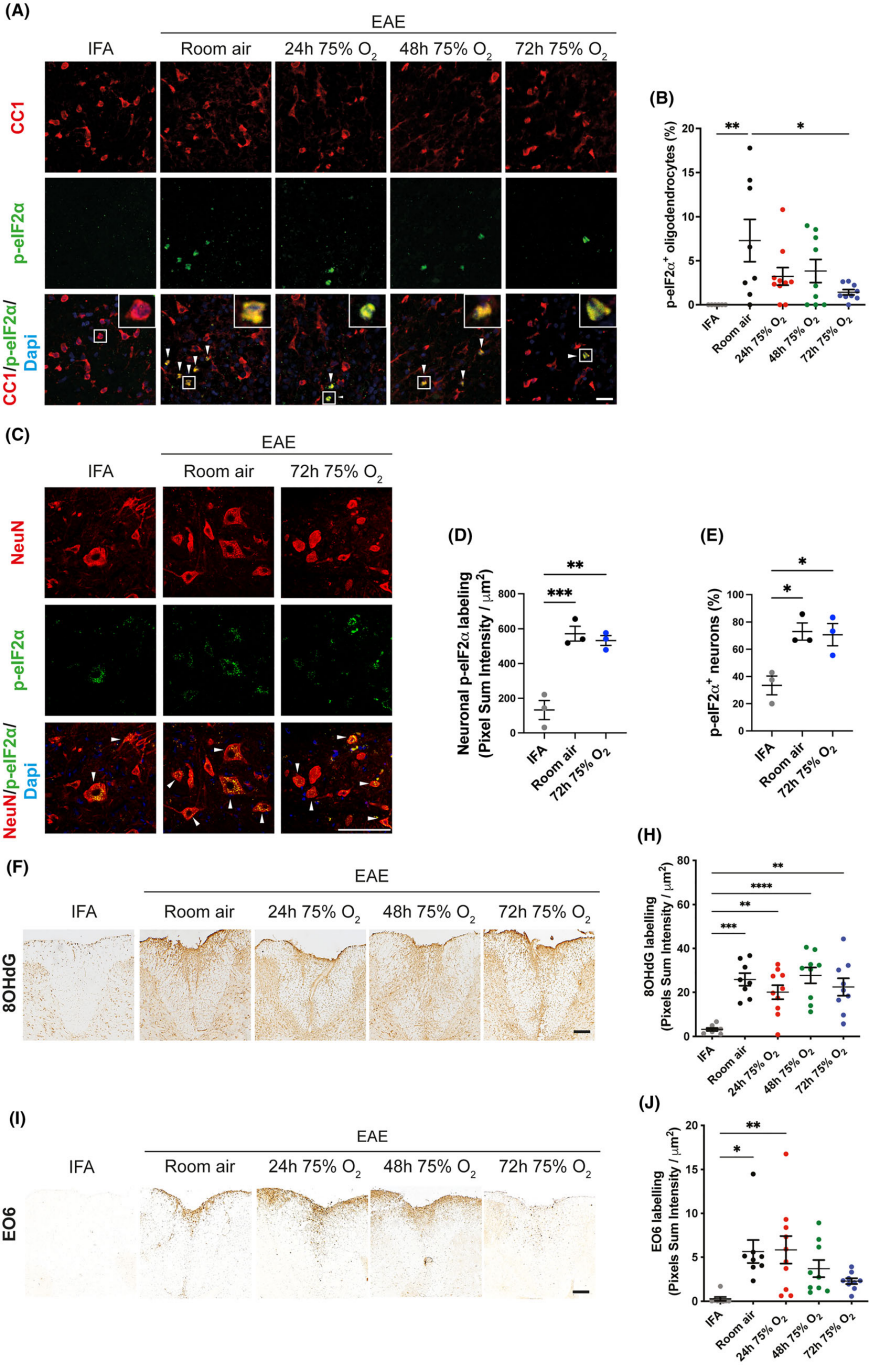
The integrated stress response in oligodendrocytes and neurons and the oxidative damage in the spinal cord of oxygen‐treated rats with active experimental autoimmune encephalomyelitis (EAE). (A) Representative images show immunofluorescence labelling for CC1 (red, oligodendrocytes), p‐eIF2α (green) and DAPI (blue, cell nuclei) in the spinal cord of incomplete Freund's adjuvant (IFA) controls and rats with active EAE at the peak of disease after exposure to room air or oxygen for either 24, 48 or 72 h. White arrowheads indicate CC1/p‐eIF2α double positive cells. High magnification insets show: absence of p‐eIF2α labelling in CC1 positive oligodendrocyte in IFA controls, but CC1/p‐eIF2α double labelling in rats with active EAE. Scale bar = 20 μm. (B) Cell counting revealed a significant increase of oligodendrocytes immunoreactive for p‐eIF2α in rats with active EAE and treated with room air compared with IFA controls. The number of CC1/p‐eIF2α double positive oligodendrocytes was decreased after treatment with oxygen, and significantly in animals exposed to oxygen for 72 h compared with room air controls. (C–E) Representative images show immunofluorescence labelling for NeuN (red, neurons), p‐eIF2α (green) and DAPI (blue, cell nuclei) in the spinal cord of IFA controls and rats with active EAE at the peak of disease after exposure to room air or oxygen for 72 h. White arrowheads indicate NeuN/p‐eIF2α double positive cells. Scale bar = 100 μm. (B) Colorimetric analysis of p‐eIF2α labelling, performed as p‐eIF2α + pixel sum intensity (or raw integrated density) normalised to NeuN area, showed increased levels of p‐eIF2α in neurons of rats with EAE compared with IFA controls. (C) Rats with EAE showed a significantly greater proportion of neurons positive for p‐eIF2α than IFA controls. No differences in neuronal p‐eIF2α levels (B) and in the proportion of NeuN/p‐eIF2α double positive neurons (C) was observed in the spinal cord or rats with EAE treated with oxygen when compared with room air controls. (F,I) Representative images show spinal cord dorsal columns labelled for 8‐OHdG (a marker of oxidised DNA and RNA) or E06 (a marker of oxidised phospholipids) in IFA controls and rats with active EAE exposed to room air or oxygen treatment for 24, 48 or 72 h from the first day of disease. Scale bar = 200 μm. (H,J) quantification of 8‐OHdG and E06 in the spinal cord white matter shows increased labelling (measured as optical density in ImageJ: integrated density/area) in rats with active EAE compared with healthy controls. The level of 8‐OHdG and E06 was not increased after oxygen administration compared with room air controls, indicating that the treatment does not exacerbate the oxidative damage. A non‐significant trend of reduced E06 labelling was observed in rats treated with oxygen for 72 h compared with room air controls. In parts (B), (H) and (J) IFA, *n* = 8; room air, *n* = 8; 24 h 75% O2, *n* = 10; 48 h 75% O2, *n* = 9; 72 h 75% O2, *n* = 9. In Parts (D) and (E), *n* = 3 per group. All statistical differences were determined using the one‐way ANOVA with Tukey's multiple comparisons test.

The phosphorylated form of the eukaryotic initiation factor‐2α (p‐eIF2α), a marker of the integrated stress response, was, as expected [44], expressed in CC1‐labelled mature oligodendrocytes of rats with active EAE, but not in IFA controls (Figure 4A). P‐eIF2α‐positive oligodendrocytes were detected in both oxygen‐treated and room air control rats, but the number of CC1/p‐eIF2α‐positive cells was significantly higher only in room air‐treated animals with active EAE compared with IFA controls (IFA vs. room air, *p* = 0.0096) and decreased in all oxygen‐treated groups, achieving significance in the group treated with oxygen for 72 h compared with room air controls (Figure 4B; room air vs. 72 h 75% O_2_, *p* = 0.025). By contrast, motor neurons showed basal expression of p‐eIF2α even in healthy conditions (IFA controls, Figure 4C). P‐eIF2α expression was increased in rats with EAE at the peak of disease (Figure 4C–E), consistent with previous findings [45, 46]. Oxygen treatment for 72 h from the onset of the disease did not change either the level of p‐eIF2α expression within neurons (Figure 4D) or the proportion of neurons positive for p‐eIF2α (Figure 4E) compared with room air controls.

#### Oxygen treatment does not exacerbate oxidative damage in rats with active EAE

As reactive inflammatory cells can produce large amounts of reactive oxygen species (ROS), we evaluated whether the administration of oxygen‐enriched air affects the severity of oxidative damage in the spinal cord of rats with active EAE. 8‐hydroxo‐2′‐deoxyguanosine (8‐OHdG, a marker for oxidised DNA and RNA) and E06 (marker of oxidised phospholipids) were histologically examined (Figure 4F–J). The spinal cord of animals with active EAE showed a significantly higher number of cells positive for 8‐OHdG and E06 compared with IFA control animals (Figure 4F–J), but there was no further increase in these markers in any of the groups treated with oxygen compared with room air controls (Figure 4H,J). Notably, while animals with EAE exposed to room air or to oxygen for 24 h had a significant increase in the level of E06 compared with IFA controls (IFA vs. room air, *p* = 0.014; IFA vs. 24 h 75% O_2_, *p* = 0.006), such an increase was not significant in the spinal cord of animals treated with oxygen for either 48 or 72 h which, on average, showed a non‐significant trend of decreased E06 levels compared with rats breathing room air (Figure 4J).

#### Spinal inflammation in rats with active EAE treated with oxygen

To investigate whether oxygen treatment affects the inflammatory response in rats with active EAE, we evaluated (1) T‐cell infiltration, (2) microglial activation and macrophage infiltration and (3) gene expression of pro‐ and anti‐inflammatory markers.

**FIGURE 5:**
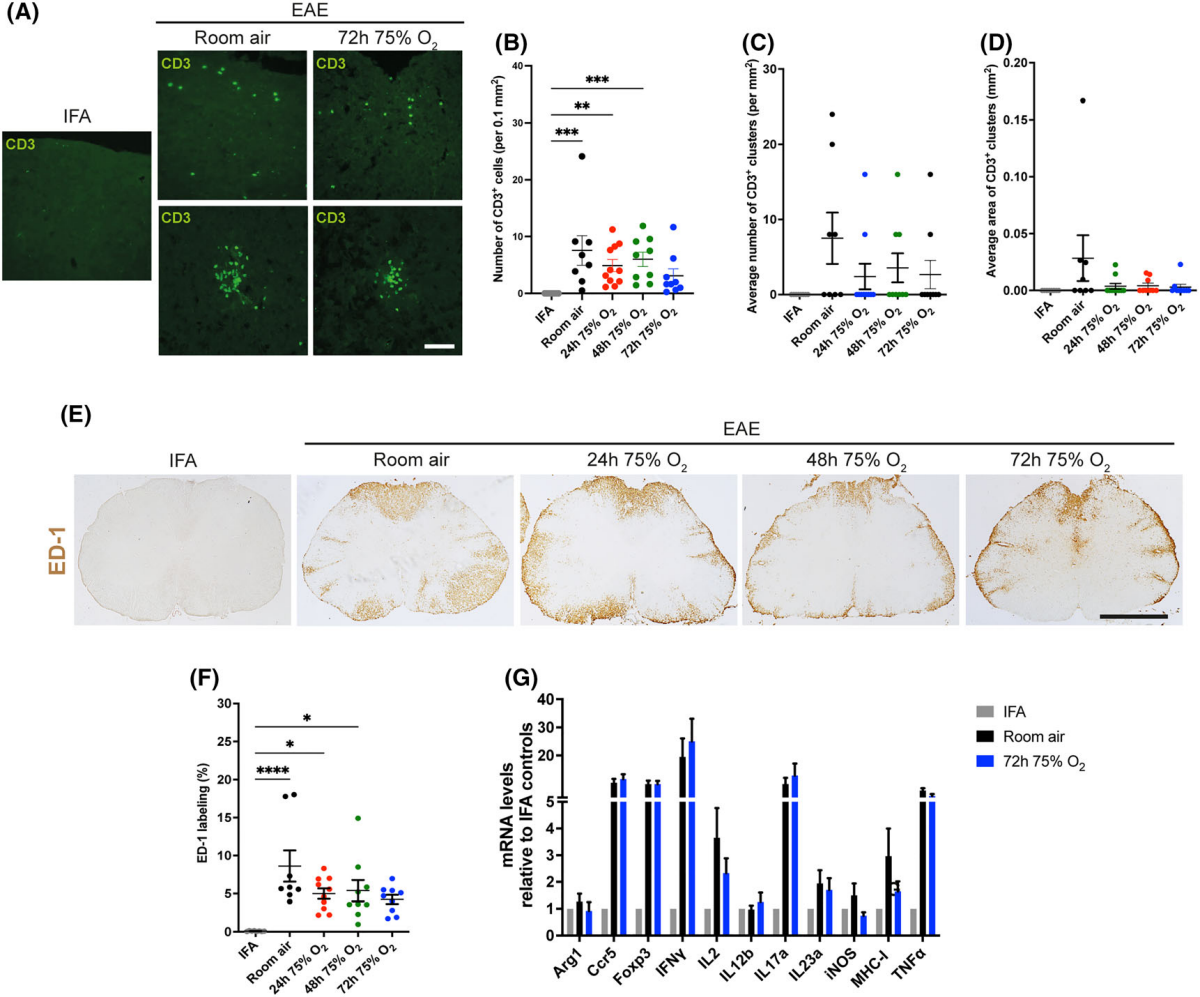
Spinal cord inflammation in rats with active experimental autoimmune encephalomyelitis (EAE) at the peak of disease, after treatment with oxygen. (A) T‐cells were labelled by immunofluorescence for CD3 (green). Representative images show isolated T‐cells and clusters of T‐cells into the spinal cord of rats with active EAE at the peak of disease, after room air or oxygen exposure for 72 h from the onset of deficits. No CD3‐positive cells were detected in the spinal cord of incomplete Freund's adjuvant (IFA) controls. Scale bar = 200 μm. (B–D) Quantification of the number of isolated CD3‐positive cells (B), or number and size of CD3‐positive clusters (C and D, respectively) showed increased CD3‐positive infiltrates in the spinal cord of animals with EAE at the peak of disease compared with healthy controls, but no changes between animals with EAE and treated with oxygen (for 24, 48 or 72 h from the onset of deficits) compared with room air controls. Kruskal–Wallis test followed by Dunn's multiple comparisons. (E) Lumbar spinal cord cross‐sections labelled for ED‐1 (brown), a marker of macrophages and activated microglia. Rats with active EAE at the peak of disease showed a remarkably stronger labelling for ED‐1 compared with IFA controls. Scale bars = 1 mm. (F) Histological quantification highlighted greater ED‐1 immunoreactivity in the spinal cord of rats with active EAE compared with healthy controls. No significant differences were observed between oxygen‐treated and room air control animals with EAE. One‐way ANOVA with Tukey's correction post‐test. (B–D,F) IFA, *n* = 8; room air, *n* = 8; 24 h 75% O2, *n* = 10; 48 h 75% O2, *n* = 9; 72 h 75% O2, *n* = 9. (G) The graph shows the fold change relative to IFA controls of the gene expression of pro‐ and anti‐inflammatory markers in the spinal cord of rats with active EAE at the peak of disease. No differences were observed between room air controls and rats treated with oxygen for 72 h from the onset of deficits; IFA, *n* = 6; EAE, *n* = 5 per group. Two‐tailed independent Student's *t* test.

Isolated CD3^+^ T‐cells, and clusters of such cells (Figure 5A), were quantified separately (Figure 5 B–D). IFA controls did not show any evidence of CD3‐positive T‐cell infiltration into the spinal cord, in contrast to animals with active EAE (Figure 5A; IFA vs. room air, *p* < 0.001; IFA vs. 24 h 75% O_2_, *p* = 0.002; IFA vs. 48 h 75% O_2_, *p* < 0.001). In rats with active EAE, oxygen treatment did not change the number of isolated CD3‐positive cells dispersed within the spinal cord parenchyma (Figure 5B) and resulted in a non‐significant decrease in the average number and size of CD3‐positive cell clusters compared with room air controls (Figure 5C,D).

At the peak of neurological deficits, animals with active EAE showed increased labelling for ED‐1‐positive activated macrophages/microglia compared with healthy controls (Figure 5E,F; IFA vs. room air, *p* < 0.0001; IFA vs. 24 h 75% O_2_, *p* < 0.03; IFA vs. 48 h 75% O_2_, *p* < 0.02). Oxygen treatment from the onset of the neurological deficit did not change ED‐1^+^ immunoreactivity compared with rats breathing room air (Figure 5E,F).

The expression of inflammatory markers in the inflamed spinal cord was analysed via quantitative RT PCR (qRT‐PCR) in rats with active EAE at the peak of deficits (3 days after onset) and treated with oxygen or room air for 72 h from disease onset (*n* = 5 per group). IFA controls (*n* = 6) were included for comparison. As expected, the qRT‐PCR analysis revealed increased levels of the pro‐inflammatory markers—Ccr5, IFNγ, IL2, IL12b, IL17a, iNOS, MHC‐I and TNFα ‐ and of the anti‐inflammatory marker FoxP3 in rats with active EAE compared with IFA controls (Figure 5G). Oxygen administration for 72 h from the onset of neurological signs did not significantly change the expression of any of these markers compared with time‐matched animals breathing room air and resulted in a non‐significant trend of decreased transcript levels of IL‐2 and MHC‐I (Figure 5G).

### Prophylactic oxygen treatment in active EAE

**FIGURE 6:**
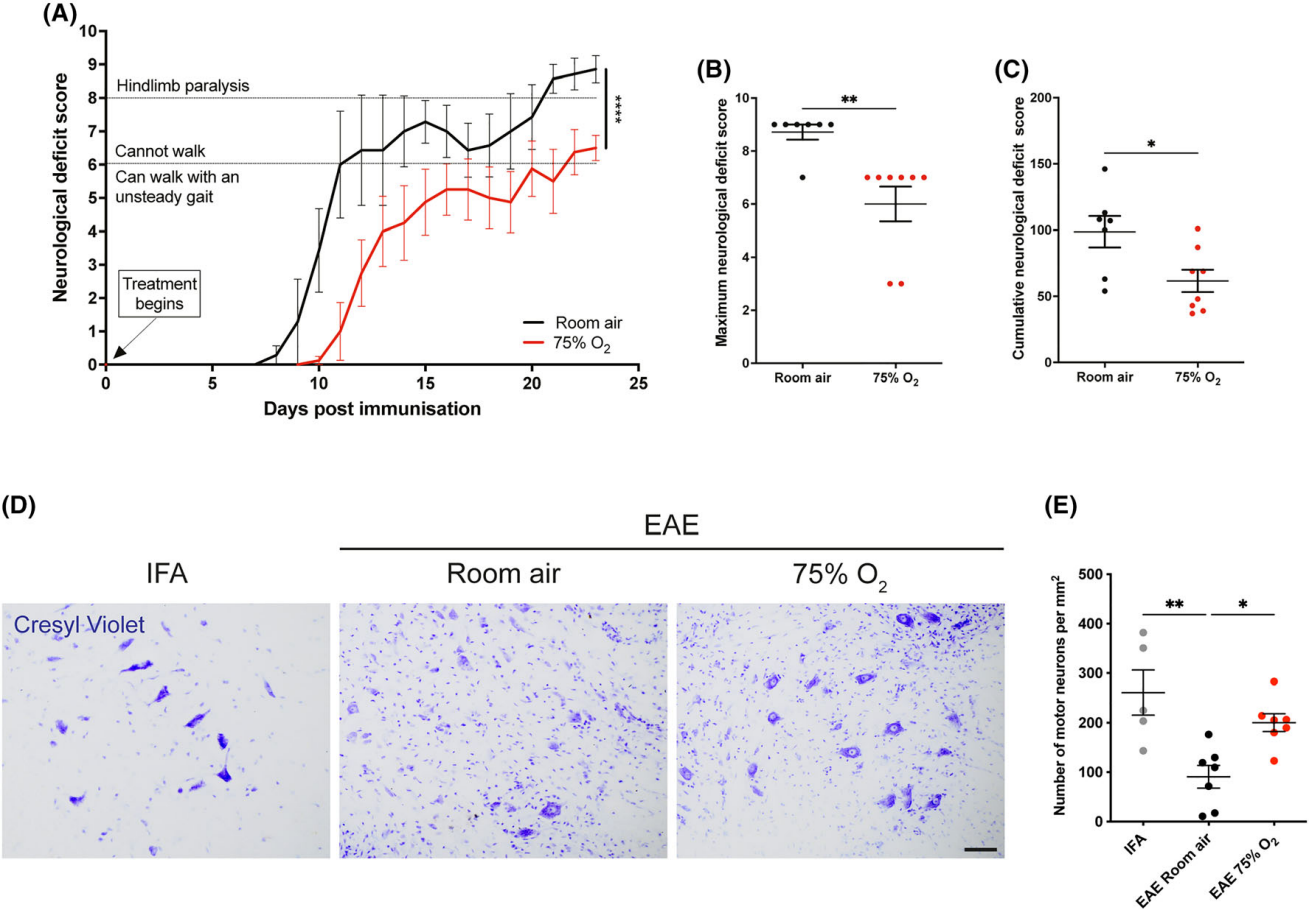
Prophylactic administration of oxygen in rats with active experimental autoimmune encephalomyelitis (EAE). (A) Oxygen treatment (75%) administered prophylactically from the day of immunisation for 23 days, delayed the onset of disease and significantly attenuated disease progression compared with room air controls. In particular, the mean score in the oxygen‐treated group was always lower than the room air control and below a score of 6, indicating that rats treated with oxygen preserved the ability to walk using their hindlimbs. Wilcoxon signed rank test; room air, *n* = 7; 75% O2, *n* = 8. (B, C) The average maximum score, and the cumulative score at the end of treatment (23 days after immunisation) reached by rats treated with oxygen prophylactically was significantly lower compared with room air controls. Two‐tailed Mann–Whitney *U* test, room air, *n* = 7; 75% O2, *n* = 8. (D) Representative images show motor neurons stained for cresyl violet (blue) in the lumbar ventral horn of the spinal cord from IFA healthy controls and rats with EAE at day 23 post‐immunisation and treated with prophylactic oxygen (75%) or room air. Scale bar = 100 μm. (E) Neuronal count showed a significant decrease in the average number of motor neurons in the ventral spinal cord of rats with EAE compared with IFA controls (*p* = 0.0019), with significant protection in rats with EAE treated prophylactically with oxygen (*p* = 0.0237). One‐way ANOVA with Tukey's multiple comparison test; IFA, *n* = 5; EAE room air, *n* = 7; EAE 75% O2, *n* = 7.

To examine whether oxygen can modify disease progression when administered prophylactically, rats with active EAE (*n* = 16) were randomly allocated to receive either oxygen (75%, *n* = 8) or room air (*n* = 8) for 23 days from the day of immunisation. Interestingly, in rats treated with oxygen the median day of onset of the neurological deficits was 2 days later (12 d.p.i.) compared with animals kept in room air (10 d.p.i.) (Figure 6A). Furthermore, prophylactic oxygen treatment significantly ameliorated disease progression compared with controls (Figure 6A; *p* < 0.0001). On average, animals exposed to oxygen maintained the ability to walk with an unsteady gait up to 22 days post‐immunisation (score ≤6) and did not develop bilateral hindlimb paralysis (score <8, Figure 6A). In contrast, animals breathing room air had already lost the ability to walk by Day 11 post‐immunisation, developing both tail and bilateral hindlimb paralysis (Figure 6A; score ≥8). The average maximum and cumulative score for neurological deficit, measures of the burden of the disease [43], were significantly lower in animals treated with oxygen compared with room air controls (maximum score; *p* = 0.001; cumulative score, *p* = 0.03) (Figure 6B,C). Thus, oxygen administration in the pre‐symptomatic phase of the disease, significantly ameliorates, and delays, the onset of neurological deficits. DA rats with active EAE are affected by spinal cord neurodegeneration at the second peak of disease. To examine whether prophylactic oxygen treatment protected from the neuronal loss due to active EAE, at the end of treatment administration (at Day 23 from immunisation, second peak of deficits), we histologically quantified the number of motor neurons in the lumbar ventral horn in spinal cord cross‐sections. As expected, rats with active EAE showed on average a lower number of motor neurons than IFA healthy controls, which was significant in rats with active EAE treated with room air (Figure 6D,E; *p* = 0.0019). Rats with active EAE treated with oxygen showed a significantly higher number of motor neurons compared with active EAE room air controls (*p* = 0.0237), suggesting that prophylactic oxygen treatment protected neurons from degeneration (Figure 6D,E; *p* = 0.0019).

### Neuropathological characterisation of rats with passive EAE

**FIGURE 7:**
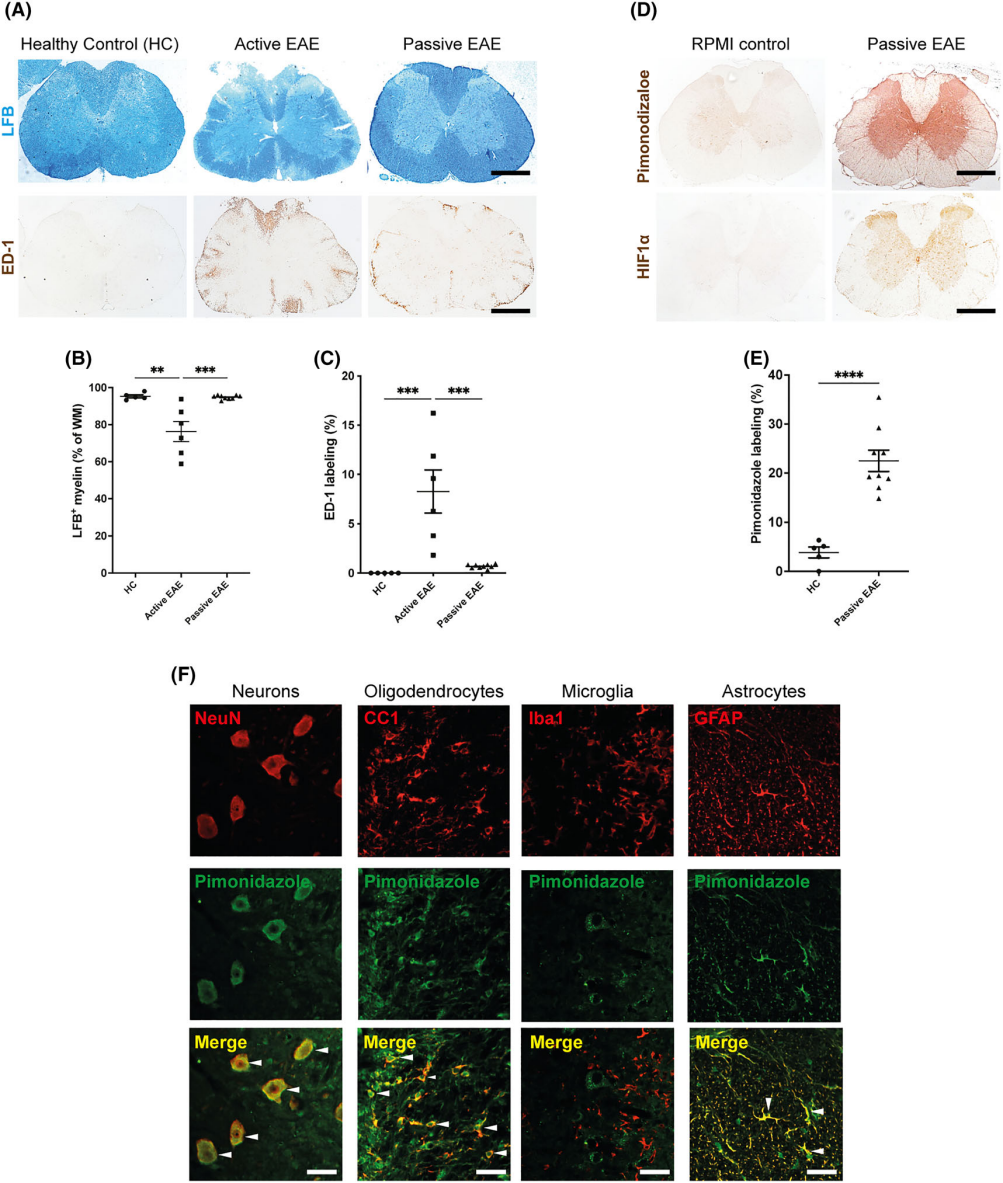
Demyelination, macroglial/macrophage activation and tissue hypoxia in the spinal cord of rats with passive experimental autoimmune encephalomyelitis (EAE). (A) Representative images of lumbar spinal cord sections stained with Luxol fast blue (LFB; blue, myelin), or ED‐1 (brown, macrophage infiltration and activated microglia) of rats with active or passive EAE at the peak of disease and healthy controls (HC, RPMI controls). Decreased myelination (loss of LFB staining) was observed in the white matter (WM) of rats with active EAE but not in passive EAE rats which showed a LFB staining pattern for LFB similar to HC. Active EAE rats also showed a greater number of spinal ED‐1 positive macrophages and microglia, compared with rats with passive EAE. Scale bars = 1 mm. (B) Quantification of LFB displayed significant loss of myelin staining in rats with active EAE, but not in HC and in rats with passive EAE. (C) Rats with active EAE showed significantly more abundant ED‐1 labelling compared with passive EAE rats and HC. (B–C) One‐way ANOVA with Tukey's multiple comparisons; HC, *n* = 5; active EAE, *n* = 6; passive EAE, *n* = 9. (D) Lumbar spinal cord cross‐sections labelled for pimonidazole and HIF1α (markers of tissue hypoxia) in rats with passive EAE and healthy controls (HC). Pimonidazole and HIF1α labelling show a similar pattern and are more intense in the spinal cord of rats with passive EAE compared with controls. Scale bars = 1 mm. (E) Histological quantification of pimonidazole showed a significant increased labelling in rats with passive EAE compared with HC. Student's *t* test; HC, *n* = 5; passive EAE, *n* = 9. (F) Immunofluorescence for pimonidazole (green) and markers of CNS cell types (red). Pimonidazole labelled the majority of neurons (NeuN), oligodendrocytes (CC1) and astrocytes (GFAP) although it was detected least commonly in microglia (Iba1). Cells with overlapping pixels (yellow) between pimonidazole (green) and CNS cell markers (red) are indicated by white arrowheads. Scale bar = 100 μm.

Histological comparison of spinal cords of rats with active and passive EAE at the peak of neurological deficits revealed several differences between these two models of MS. Luxol fast blue (LFB) staining at the peak of disease revealed a prominent loss of myelin in the white matter of animals with active EAE, whereas demyelination was absent in animals with passive EAE (Figure 7A,B; HC vs. active EAE, *p* = 0.001; active EAE vs. passive EAE, *p* = 0.0005). Furthermore, at the peak of disease, labelling for ED‐1‐positive activated macrophages/microglia was significantly lower in rats with passive EAE compared with animals with active EAE (Figure 7A,C; HC vs. active EAE, *p* = 0.0006; active EAE vs. passive EAE, *p* = 0.0003). In passive EAE, ED‐1‐positive cells were mainly evident in the superficial white matter and around a few blood vessels, and these cells were not spread in the CNS parenchyma as observed in active EAE (Figure 7A).

### Hypoxia in the spinal cord of rats with passive EAE

To determine whether hypoxia is a feature of passive EAE (*n* = 9), we examined spinal cord tissue histologically for evidence of pimonidazole adducts [47, 48] and HIF1α. Labelling for pimonidazole was prominent in the spinal cord of animals with passive EAE (Figure 7D). Moreover, labelling for HIF1α also showed a similar pattern (Figure 7D). Quantification of pimonidazole labelling revealed a significantly greater extent of labelling in the spinal cord of animals expressing a neurological deficit, compared with RPMI‐treated healthy controls (Figure 7D; *p* < 0.0001). Qualitative inspection at higher magnification revealed strong, cell‐specific labelling and double immunofluorescent labelling revealed pimonidazole adducts in the majority of NeuN‐positive neurons, CC1‐positive oligodendrocytes and GFAP‐positive astrocytes, but in only a few Iba1‐positive macrophages/microglia (Figure 7F). In addition, *live* measurements of the oxygen tension in the spinal cord grey matter of anaesthetized rats showed a trend (*p* = 0.0759) of decreased oxygen concentration in rats with passive EAE (at the peak of disease, score 5) compared with RPMI‐treated healthy controls (Figure S1A,B). The magnitude of the decrease in spinal oxygen concentration in rats with passive EAE was comparable with the one we observed in a previous study [4] in rats with active EAE and the same score of neurological deficit (score 5).

### Oxygen therapy in passive EAE

**FIGURE 8:**
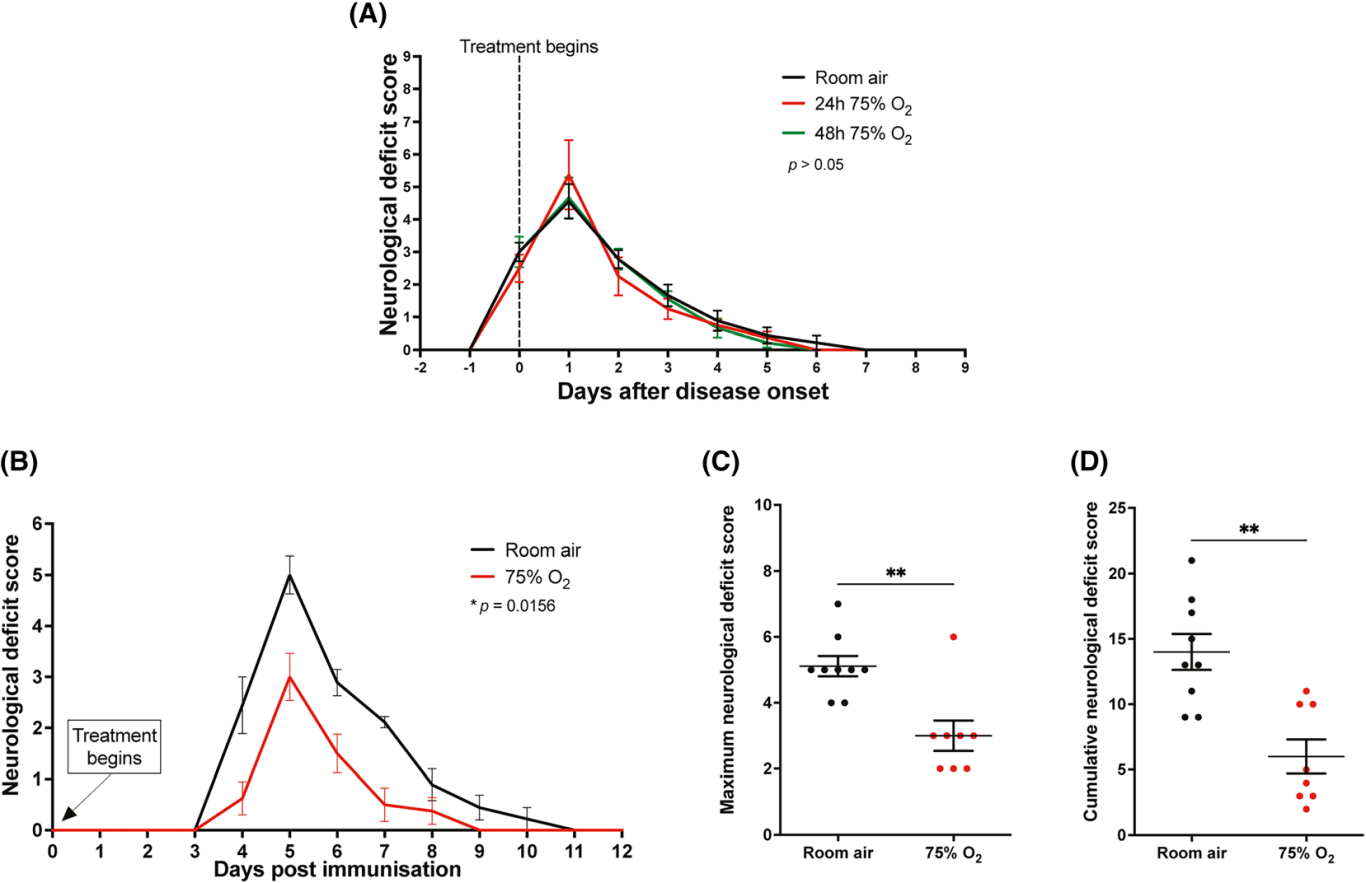
Oxygen treatment in rats with passive experimental autoimmune encephalomyelitis (EAE). (A) Rats with passive EAE were kept in room air or treated with oxygen (75%) from the day of onset of neurological deficit for either 24 or 48 h. After oxygen treatment, rats were returned to room air and monitored up to 4 days after the complete remission of the disease. No changes in disease severity were observed between oxygen‐treated rats and room air controls. Room air, *n* = 9; 24 h 75% O2, *n* = 8; 48 h 75% O2, *n* = 9. The clinical progression of EAE between groups was compared using the repeated measurements Friedman's one‐way ANOVA with Dunn's multiple comparisons test (*p* > 0.05). (B) Rats with passive EAE were kept in room air or treated with oxygen (75%) from the day of immunisation up to 4 days after complete remission of the disease. Prophylactic oxygen treatment significantly ameliorated the severity of neurological deficits compared with room air controls. Wilcoxon signed rank test; room air, *n* = 9; 75% O2, *n* = 8. (C,D) The average maximum score (C), and the cumulative neurological deficit score (D) reached by rats treated with oxygen prophylactically was significantly lower compared with room air controls. Statistical differences were determined using the two‐tailed Mann–Whitney *U* test. Room air, *n* = 9; 75% O2, *n* = 8.

To assess whether oxygen could provide benefit in passive EAE in DA rats (*n* = 35), oxygen treatment was administered therapeutically for 24 or 48 h from the onset of the deficit (room air, *n* = 9; 24 h 75% O_2_, *n* = 8; 48 h 75% O_2_, *n* = 9) or prophylactically (room air, *n* = 8; prophylactic 75% O_2_, *n* = 8). Following treatment with oxygen, animals were returned to room air and the neurological deficits were monitored up to the fourth day after the complete recovery of the disease. In contrast with active EAE, oxygen administration commencing at the onset of deficits for 24 and 48 h did not ameliorate neurological dysfunction compared with room air controls, and the disease course was similar between treated and untreated animals (Figure 8A). However, when oxygen was administered prophylactically, it improved neurological function compared with room air controls (Figure 8B; *p* = 0.016), resulting in a significant reduction of both maximum neurological deficit score (*p* = 0.004) and cumulative score (*p* = 0.003) (Figure 8C,D). Prophylactic treatment with oxygen did not delay the onset of the disease.

## DISCUSSION

The results show that breathing air with raised oxygen can promptly reduce the neurological deficits, demyelination and oligodendrocyte death associated with autoimmune neuroinflammatory disease, without increasing oxidative damage. The neurological deficits are not only promptly reduced upon breathing oxygen but also promptly restored by breathing room air. However, continued (1–3 days) therapy provides prolonged protection that persists after return to room air. Therapy is most beneficial when applied early after the onset of neurological deficits, and the greatest beneficial histological benefits were observed in rats treated for 72 h from the onset of neurological deficit.

Oxygen has many biological effects, but the prompt improvement in neurological deficits upon oxygen administration in active EAE (see also Davies et al. [4]), and its prompt reversal upon return to room air, focuses attention on mitochondrial function as the most likely mediator of the effects. Mitochondrial dysfunction has been identified as a major cause of reversible neurological deficits in neuroinflammatory disease [31, 32], and oxygen deprivation is a prominent cause of the mitochondrial and axonal depolarization known to parallel the expression of deficits at the onset of disease and during relapse: the depolarization improves during remission in conjunction with the deficit [32]. The most likely cause of the oxygen deprivation is hypoperfusion of the inflamed tissue, which is prominent in both EAE [5, 49] and MS [7, 8, 9, 11, 12, 18].

The significant reduction in demyelination mediated by oxygen is unexpected given that demyelination in active EAE is believed to be mediated by anti‐MOG antibodies [50, 51] and comparable with the Patterns I and II demyelination described by Lucchinetti et al. in MS [52]. The current finding suggests that, in addition to antibody‐mediated demyelination, some demyelination in autoimmune rMOG EAE is promoted by the profound tissue hypoxia [4] and is akin to the Pattern III demyelination known to be caused by hypoxia [5, 53].

A role for tissue hypoxia has been described in regulating the pro‐inflammatory activation of immune cells [54]. In particular, the HIF1α pathway has been shown to drive the differentiation of naïve CD4 T‐cells into T helpers, supporting the induction of EAE after immunisation with MOG [55]. Therefore, the later onset of deficits observed in rats with active EAE treated with prophylactic oxygen raised the possibility that oxygen therapy may have played an immunomodulatory effect on the priming and expansion of the disease‐initiating, autoreactive CD4 T‐cells. Interestingly, we observed improved neurological deficits also in rats with passive EAE after treatment with prophylactic oxygen, arguing against an immunomodulatory beneficial effect of oxygen, at least during the induction phase of the disease. This is because, in passive EAE, CD4 T‐cells are reactivated in vitro and so treatments cannot affect the initial activation or proliferation of the encephalitogenic T‐cells once injected in the recipient animal [56]. This feature makes the passive EAE model a valuable tool to assess whether the efficacy of a treatment relies or not on the priming of the autoimmune response [57, 58].

Although we did not detect significant changes in the number of T‐cells and in the gene expression of pro‐inflammatory markers in the spinal cord from rats with active EAE after treatment with oxygen, we cannot entirely rule out more subtle effects that oxygen may have on the inflammation.

From the current findings, we suppose that oxygen plays its beneficial role through metabolic support to the CNS rather than via an anti‐inflammatory effect. However, we recommend further investigations, using more sensitive techniques such as flow cytometry and ELISA assays, to elucidate whether oxygen therapy affects the migration, differentiation, and/or function of immune cells during EAE.

The protective effect on demyelination may have been a consequence of the decreased integrated stress response in oligodendrocytes, highlighted by a reduction in eIF2α phosphorylation. Tissue hypoxia has been commonly associated with the phosphorylation of eIF2α in a cellular attempt to save energy by decreasing global protein synthesis and selectively allowing the translation of protective proteins [59, 60, 61]. Although protective at first, prolonged phosphorylation of eIF2α can trigger apoptosis [62]. Until now, eIF2α phosphorylation in oligodendrocytes during active EAE has been mostly attributed to the cytotoxic impact of inflammation and in particular to increased levels of IFNγ [63]. Our findings demonstrate that oxygen is able to decrease eIF2α phosphorylation without changing the level of IFNγor other inflammatory markers, suggesting that in active EAE also hypoxia can be a source of cellular stress in oligodendrocytes. We believe that by decreasing the phosphorylation of eIF2α, oxygen therapy may have helped the oligodendrocytes to escape the apoptotic pathway, ultimately protecting the spinal cord from demyelinating.

Interestingly, prophylactic treatment with oxygen of rats with active EAE showed the greatest beneficial effect on neurological deficits and protected from neuronal loss at the second peak of the disease. Understanding whether the neuroprotective effect was a consequence of reduced demyelination or due to a direct effect of oxygen on neuronal survival, for example, by improving the mitochondrial function in neurons, will be the subject of future investigations.

A potential safety concern for the use of oxygen as a treatment in MS is the fact that increased oxygenation can enhance oxidative stress and damage and yet we found that oxygen exposure from the onset of deficits did not exacerbate oxidative stress at the peak of disease compared with room air controls. Instead, when oxygen was administered for 72 h, there was a trend towards decreased levels of oxidised phospholipids compared with room air controls and rats treated with oxygen for 24 or 48 h. A reduction is not entirely unexpected because hypoxia, as well as hyperoxia, can enhance the production of mitochondrial reactive oxygen species [64, 65], and so raising the oxygen concentration in a tissue can also reduce oxidative stress.

The neurological deficit in animals with passive EAE can be severe, but there is much less pathology and so the cause of the deficit has been unclear. Our finding that the spinal cord labels for hypoxia and the expression of HIF1α raises suspicion that the deficit may be due to hypoxia‐induced mitochondrial failure and this interpretation is supported by the significant reduction in neurological deficit when hypoxia was avoided by prophylactic treatment with oxygen. The absence of protection when oxygen administration was delayed until after the onset of neurological deficit is interesting and adds to the evidence from active EAE that early treatment is most beneficial. The particularly fast progression of the neurological deficits in passive EAE may have narrowed the therapeutic window for oxygen to be effective.

The current findings raise the long and vexed history of oxygen as a therapy in MS. Although many patients report that oxygen therapy reliably provides a temporary (days) reduction in symptoms such as fatigue and bladder dysfunction, such therapy is discounted by many neurologists, who are supported by Cochrane and other assessment of clinical trials [66, 67, 68]. The sceptical opinion has been bolstered by a belief that any beneficial effects of oxygen therapy are ‘difficult to ascribe with biological plausibility’, but we believe that such plausibility is now provided by both the findings of tissue hypoxia in neuroinflammatory disease [4, 5, 28, 29, 30, 53, 69], and the clearly beneficial effects of oxygen therapy in experimental models (current study and other studies [4, 5, 53]). However, there are several important differences between the current observations and oxygen therapy in MS, not least the treatment dose (normobaric vs. hyperbaric), and duration (days vs. hour[s]), meaning that the current findings and the hyperbaric trials are not directly comparable.

## CONCLUSIONS

The current findings encourage exploration of the value of normobaric oxygen administered early after the onset of new neurological deficits that herald a relapse in MS, with the aim of improving the oxygenation of the inflamed tissue. Such therapy may be expected not only to improve neurological function but also to reduce demyelination. Therapy with similar goals has been advanced using the CNS selective vasodilating drug nimodipine [5], and such therapeutic approaches may be valuable adjuncts to immunomodulatory therapies, especially during relapses.

## CONFLICT OF INTEREST

The authors have no conflicts of interest to disclose.

## ETHICS STATEMENT

Studies using rats were approved by the IACUC at the UCL Institute of Neurology and adhered to the UK Animals (Scientific Procedures) Act of 1986, and the ARRIVE guidelines.

## AUTHOR CONTRIBUTIONS

Mario Amatruda, Andrew L. Davies, Andrew L. Davies, and Kenneth J. Smith conceptualised and designed the study. Mario Amatruda was responsible for methodology, validation, acquisition of data, investigation and formal analysis. Mario Amatruda, Kate Harris, Alina Matis, and Michael Clark performed histological analyses. Daniel McElroy and Christopher Linington provided the encephalitogenic T‐cells and performed the qRT‐PCR. Mario Amatruda, Kate Harris, Alina Matis, and Andrew L. Davies were responsible for the blinded behavioural assessments. Roshni Desai performed pimonidazole injections in the saphenous vein. Mario Amatruda wrote the original draft. Mario Amatruda, Roshni Desai, and Kenneth J. Smith reviewed and edited the manuscript. Kenneth J. Smith acquired the funding. All co‐authors critically reviewed the manuscript for content.

### PEER REVIEW

The peer review history for this article is available at https://publons.com/publon/10.1111/nan.12868.

## Supporting information


**Table S1.** **Primary antibodies.** Antibodies were used on rat tissue for immunohistochemistry and/or immunofluorescence as described in the material and methods. mAb = monoclonal antibody, pAb = polyclonal antibody.

**Table S2.** **RT‐PCR primers and conditions.** Abbreviations are: FW = forward primer; RV = reverse primer.

**Figure S1.** **In vivo oxygen concentration within the grey matter of rats with passive EAE. (A)** Representative records obtained from the insertion (downward arrow) and then withdrawn (upward arrow) of an oxygen probe into the dorsal horn of the spinal cord grey matter of an anaesthetised rat with passive EAE (Passive EAE, red line) and an RPMI healthy control (black line). **(B)** Quantification of the average oxygen probe measurements, obtained during the stable recording period, shows a trend of decreased oxygen tension in the spinal cord of rats with passive EAE compared with healthy controls (*p* = 0.0759, student *t* test). Healthy control, n = 7; passive EAE, n = 8.

## Data Availability

The data that support the findings of this study are available from the corresponding author, upon reasonable request.
