# Supplementary material for: Oxygen treatment reduces neurological deficits and demyelination in two animal models of multiple sclerosis
Source: Neuropathol Appl Neurobiol. 2023 Jan 10;49(1):e12868. doi: 10.1111/nan.12868 (PMC10107096; doi:10.1111/nan.12868)
Supplement: Supplementary file 1 — Table S1. Primary antibodies. Antibodies were used on rat tissue for immunohistochemistry and/or immunofluorescence as described in the material and methods. mAb = monoclonal antibody, pAb = polyclonal antibody. [file NAN-49-0-s003.docx]

**Supplementary Table 1. Primary antibodies.** Antibodies were used on rat tissue for immunohistochemistry and/or immunofluorescence as described in the material and methods. mAb = monoclonal antibody, pAb = polyclonal antibody.

| **Antibody** | **Origin** | **Target** | **Marker** | **Dilution** | **Source** |
| --- | --- | --- | --- | --- | --- |
| **8OHdG** | Mouse (mAb) | 8-hydroxy-2'-deoxyguanosine | Oxidized DNA/RNA, oxidative damage | 1/200 | Trevigen; 4354MC050 |
| **CC1** | Mouse (mAb) | Adenomatous poli coli protein,clone CC1 | Oligodendrocyte cell body | 1/200 | Abcam; ab16794 |
| **CD3** | Mouse (mAb) | Cluster of differentiation 3 | T-lymphocytes | 1/200 | AbD Serotec;  MCA772 |
| **E06** | Mouse (mAb) | Oxidized phospholipids | Oxidative damage | 1/200 | Avanti; 330001 |
| **ED-1** | Mouse (mAb) | Rat CD68 | Activated microglia and macrophages | 1/200 | AbD Serotec; MCA341G |
| **GFAP** | Rabbit (pAb) | Glial fibrillary acidic protein | Astrocytes | 1/1000 | Dako; Z0334 |
| **HIF1α** | Rabbit (pAB) | Hypoxia inducible factor-1α | Tissue hypoxia | 1/200 | Abcam; ab85886 |
| **Hydroxyprobe-1-anti-pimonidazole** | Mouse (mAb) Rabbit (pAB) | Pimonidazole (Pimo) adducts | Hypoxia | 1/500 | HPI Inc. |
| **Iba1** | Rabbit (pAb) | Ionised calcium binding adaptor-1 | Microglia | 1/500 | Wako;  019-19741 |
| **MOG** | Mouse (mAb) | Myelin oligodendrocyte glycoprotein | Myelin | 1/500 | Millipore; MAB5680 |
| **NeuN** | Mouse (mAb) | Neuronal nuclei | Neuronal cell body | 1/200 | Millipore; MAB377 |
| **p-eIF2α** | Goat (pAb) | Phosphorylated eukaryotic initiation factor-2α | Integrated stress response | 1/200 | Santa Cruz; sc12412 |
