# Supplementary material for: Oxygen treatment reduces neurological deficits and demyelination in two animal models of multiple sclerosis
Source: Neuropathol Appl Neurobiol. 2023 Jan 10;49(1):e12868. doi: 10.1111/nan.12868 (PMC10107096; doi:10.1111/nan.12868)
Supplement: Supplementary file 2 — Table S2. RT‐PCR primers and conditions. Abbreviations are: FW = forward primer; RV = reverse primer. [file NAN-49-0-s002.docx]

**Supplementary Table 2. RT-PCR primers and conditions.** Abbreviations are: FW = forward primer; RV = reverse primer.

| **Gene** | **Full name** | **Primer sequences** | **Size (bp)** | **Annealing (°C)** | **Gene bank** |
| --- | --- | --- | --- | --- | --- |
| **Arg1** | Arginase-1 | FW: 5’-CATATCTGCCAAGGACATCG-3’  RV: 5’-GGTCTCTTCCATCACTTTGC-3’ | 142 | 56 | NM_017134.3 |
| **Ccr5** | C-C chemokine receptor 5 | FW: 5’-GAAGGTGAGACATCCGTTCCC-3’  RV: 5’-CAGACGTCCCCTCAGGATTTC-3’ | 126 | 60 | NC_005107.4 |
| **Foxp3** | Forkhead box P3 | FW: 5’-TTCTCAAGCACTGCCAAGCA-3’  RV: 5’-GTCTCCGCACAGCAAACAAG-3’ | 291 | 60 | NM_001108250.1 |
| **IFNγ** | Interferon-γ | FW: 5’-GCCCTCTCTGGCTGTTACTG-3’  RV: 5’-CCAAGAGGAGGCTCTTTCCT-3’ | 112 | 60 | NM_138880.2 |
| **IL-2** | Interleukine-2 | FW: 5’-CCAAGCAGGCCACAGAATTG-3’  RV: 5’- ACCACAGTTGCTGGCTCATC-3’ | 208 | 60 | NM_053836.1 |
| **IL-12b** | Interleukine-12b | FW: 5’-ACCTCTGCCGAAGTCCAATG-3’  RV: 5’-AAGCCTCCTCTTAGCCTCCA-3’ | 172 | 60 | NC_005109.4 |
| **IL-17a** | Interleukine-17a | FW: 5’-CATCCATGTGCCTGATGCTG-3’  RV: 5’-GCCTCCCAGATCACAGAAGG-3’ | 265 | 59 | NM_001106897.1 |
| **IL-23** | Interleukine-23 | FW: 5’-ATGACCAGCTTCCTTCCACC-3’  RV: 5’-TCACCTCACCTCGTTGTTCC-3’ | 198 | 60 | NC_005106.4 |
| **iNOS** | Inducible nitric oxide synthase | FW: 5’-GAGACGCACAGGCAGAGG-3’  RV: 5’-CAGGCACACGCAATGATGG-3’ | 121 | 60 | NM_012611.3 |
| **Mhc1** | Major histocompatibility complex-1 | FW: 5’-TGACCAACCTTTCCACAGCAT-3’  RV: 5’-CAAGACCAGAGCATCACACCA-3’ | 198 | 60 | NC_005112.4 |
| **TNFα** | Tumor necrosis factor-α | FW: 5’-CCCCTTTATCGTCTACTCCTCA-3’  RV: 5’-TTCAGCGTCTCGTGTGTTTC-3’ | 134 | 58 | NM_012675.3 |
