# Supplementary figures and images for: Oxygen treatment reduces neurological deficits and demyelination in two animal models of multiple sclerosis
Source: Neuropathol Appl Neurobiol. 2023 Jan 10;49(1):e12868. doi: 10.1111/nan.12868 (PMC10107096; doi:10.1111/nan.12868)

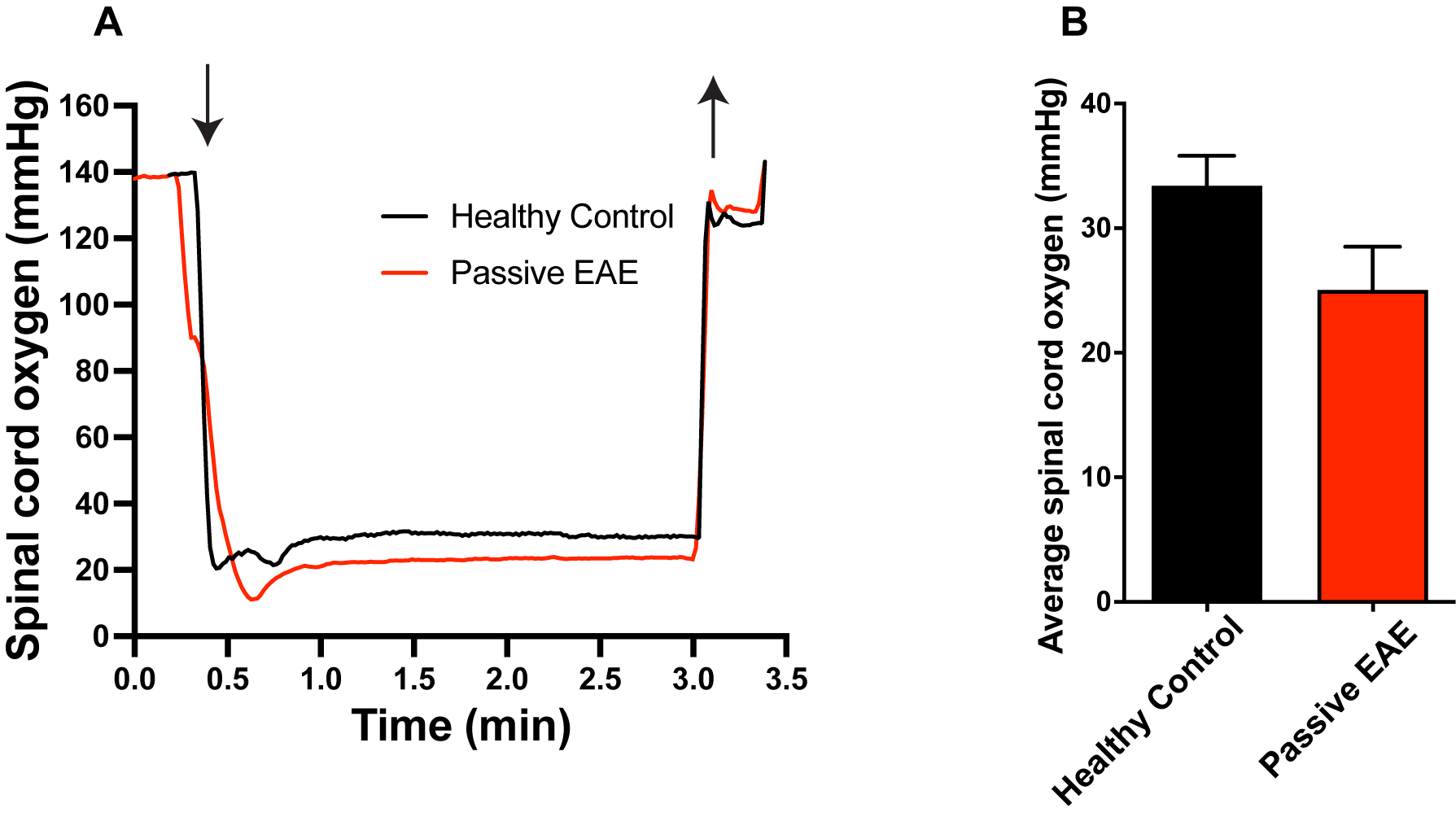

Supplement: Supplementary file 3 — Figure S1. In vivo oxygen concentration within the grey matter of rats with passive EAE. (A) Representative records obtained from the insertion (downward arrow) and then withdrawn (upward arrow) of an oxygen probe into the dorsal horn of the spinal cord grey matter of an anaesthetised rat with passive EAE (Passive EAE, red line) and an RPMI healthy control (black line). (B) Quantification of the average oxygen probe measurements, obtained during the stable recording period, shows a trend of decreased oxygen tension in the spinal cord of rats with passive EAE compared with healthy controls (p = 0.0759, student t test). Healthy control, n = 7; passive EAE, n = 8. [file NAN-49-0-s001.tif]
